# Cytostatic potential of novel agents that inhibit the regulation of intracellular pH

**DOI:** 10.1038/sj.bjc.6600424

**Published:** 2002-07-02

**Authors:** P Wong, H-W Kleemann, I F Tannock

**Affiliations:** Department of Medical Biophysics, Princess Margaret Hospital/Ontario Cancer Institute, University of Toronto, Toronto, M5G 2M9, Canada; Aventis Pharma Deutschland GmbH, Industriepark Höchst, 65926 Frankfurt/Main, Germany; Department of Medical Oncology and Hematology and the Division of Experimental Therapy, Princess Margaret Hospital, University of Toronto, Toronto, M5G 2M9, Canada

**Keywords:** cariporide, S3705, Na^+^/H^+^ antiport, Na^+^-dependent Cl^−^/HCO_3_^−^ exchanger, intracellular pH

## Abstract

Cells within the acidic extracellular environment of solid tumours maintain their intracellular pH (pHi) through the activity of membrane-based ion exchange mechanisms including the Na^+^/H^+^ antiport and the Na^+^-dependent Cl^−^/HCO_3_^−^ exchanger. Inhibition of these regulatory mechanisms has been proposed as an approach to tumour therapy. Previously available inhibitors of these exchangers were toxic (e.g. 4,4-diisothiocyanstilbene-2,2-disulphonic acid), and/or non-specific (e.g. 5-N-ethyl-N-isopropyl amiloride). Using two human (MCF7, MDA-MB231) and one murine (EMT6) breast cancer cell lines, we evaluated the influence of two new agents, cariporide (an inhibitor of the Na^+^/H^+^ antiport) and S3705 (an inhibitor of the Na^+^-dependent Cl^−^/HCO_3_^−^ exchanger) on the regulation of intracellular pH (pHi). The cytotoxicity of the two agents was assessed by using clonogenic assays. Our results suggest that cariporide has similar efficacy and potency to 5-N-ethyl-N-isopropyl amiloride for inhibition of Na^+^/H^+^ exchange while S3705 is more potent and efficient than 4,4-diisothiocyanstilbene-2,2-disulphonic acid in inhibiting Na+-dependent Cl^−^/HCO3^−^ exchange. The agents inhibited the growth of tumour cells when they were incubated at low pHe (7.0–6.8), but were non-toxic to cells grown at doses that inhibited the regulation of pHi. Our results indicate that cariporide and S3705 are selective cytostatic agents under *in vitro* conditions that reflect the slightly acidic microenvironment found in solid tumours.

*British Journal of Cancer* (2002) **37**, 238–245. doi:10.1038/sj.bjc.6600424 www.bjcancer.com

© 2002 Cancer Research UK

## 

The microenvironment within many solid tumours is known to be acidic (mean extracellular pH (pHe) ≅ 6.9–7.0) as compared to normal tissues (mean pHe ≅ 7.4). The acidity in solid tumours is due to the increased production of acidic metabolites such as lactic acid and CO_2_ and to the limited ability of the tumour vasculature to remove these acidic products of metabolism, which therefore accumulate within the tumour microenvironment. Using a pH-sensitive fluorescent probe and fluorescent microscopy applied to a solid tumour growing in a transparent window, Helmlinger *et al* (1997) demonstrated a gradual decrease of pHe from 7.4 to 6.7 as the distance from blood vessels increased from 0 μM to 200 μM.

Under acidic conditions, cells regulate their pHi by buffering protons that enter the cell, and by activating membrane-based ion-exchange mechanisms, of which the most important are the Na^+^/H^+^ antiport and the Na^+^-dependent HCO_3_^−^/Cl^−^ exchanger. While the intracellular buffering capacity serves to minimize the change in pHi during minor influx or efflux of H^+^ or OH^−^, restoration of homeostasis is achieved by activating the membrane based ion-exchange mechanisms (Murer *et al*, 1976; Thomas, 1977; Tonnessen *et al*, 1990).

Na^+^/H^+^ antiports are expressed in all mammalian cells. Six isoforms of the Na^+^/H^+^ antiports have been identified and named NHE1–6 (Sardet *et al*, 1989; Orlowski *et al*, 1992; Tse *et al*, 1992; Tse *et al*, 1993; Wang *et al*, 1993; Klanke *et al*, 1995; Numata *et al*, 1998). While NHE1 is found in most cells, NHE2-5 are tissue specific and NHE6 is expressed in mitochondria (Counillon and Pouyssegur, 2000). The ubiquitous pattern of expression of NHE1 suggests that it is the dominant form that cells use to regulate their cell volume and pHi. When NHE1 is activated by intracellular acidosis, it exports excess H^+^ ions from the cytoplasm to the extracellular environment in exchange for the intake of Na^+^ ions with a 1 : 1 stoichiometry (Grinstein *et al*, 1984; Aronson, 1985).

The gene that encodes the Na^+^-dependent HCO_3_^−^/Cl^−^ exchanger was cloned and named NCBE (Wang *et al*, 2000). NCBE regulates pHi by importing HCO_3_^−^ from the extracellular space into the cell to buffer excess protons, thus inhibiting changes in pHi. The activity of NCBE depends on the presence of extracellular Na^+^ and HCO_3_^−^, and on intracellular Cl^−^ ions.

Our laboratory and others have evaluated previously the efficacy of available NHE-1 inhibitors, such as amiloride and its analogue 5-N-ethyl-N-isopropyl amiloride (EIPA) with the goal of assessing their possible role in the treatment of solid tumours, where they might contribute to cellular toxicity under acidic conditions. These agents are quite effective inhibitors of Na^+^/H^+^ exchange but lack specificity (Kleyman and Cragoe, 1988; Newell *et al*, 1992; Horvat *et al*, 1993; Murata *et al*, 1995). Despite their lack of specificity, experimental studies using amiloride and EIPA have suggested that inhibition of NHE-1 might be beneficial to patients with myocardial ischemia (Pierce *et al*, 1993; Baumgarth *et al*, 1996). A new inhibitor, cariporide (HOE642) has been developed to inhibit specifically the NHE-1 isoform (Scholz *et al*, 1995) by competing with Na^+^ for its binding site. Phase II/III clinical trials of cariporide have been completed and suggest that this agent provides patients with protection from cardiac ischemia and reperfusion injury (Karmazyn, 2000; Theroux *et al*, 2000).

Previously, inhibitors of Na^+^-dependent Cl^−^/HCO_3_^−^ exchange were limited to stilbene derivatives such as 4,4-diisothiocyanstilbene-2, 2-disulphonic acid (DIDS). This agent provides partial non-reversible inhibition of the exchanger and is toxic to cells *in vitro* at 0.4 mM and quite toxic i*n vivo* (Yamagata and Tannock, 1996). More recently, investigators from the Aventis Pharmaceutical Company have developed a new inhibitor of the Na^+^-dependent Cl^−^/HCO3^−^ exchanger, known as S3705 (unpublished data).

Under acidic conditions, proliferation of cells is known to be dependent on the pH regulatory mechanisms to maintain their intracellular pH within the range of pHi 7.2-7.4 (Rotin *et al*, 1989). If NHE-1 and NCBE are inhibited, the pHi of cells might be expected to establish equilibrium with the extracellular pH (pHe), which would inhibit selectively the metabolism and growth of tumour cells that are in an acidic microenvironment (Bock and Frieden, 1976; Busa and Nuccitelli 1984; Musgrove *et al*, 1987). Therefore, cariporide and S3705 might have cytostatic effects on cells growing under the acidic conditions that are found in solid tumours although this will depend on the ability of these agents to penetrate tissue to reach these acidic cells. Cells in these regions may be relatively resistant to chemotherapy due to poor drug access, and agents that inhibit the proliferation of cells in acidic regions of tumours might have therapeutic value when used in conjunction with chemotherapy.

In the present article, we characterise the activity of cariporide and S3705 as inhibitors of their respective exchangers, the Na^+^/H^+^ antiport and the Na^+^-dependent Cl^−^/HCO_3_^−^ exchanger, in cultured malignant cells. We measure the pHi and the rate of pHi recovery after intra-cellular acidification. Finally, we assess the ability of these agents to inhibit cell proliferation and to cause cell death, as a function of pHe.

## METHODS

### Cells

Experiments were performed using murine EMT-6 cells derived from a mammary sarcoma, and two human breast cancer cell lines, MCF7 and MDA-MB231 (both purchased from the American Type Culture Collection). Cells were maintained in α-Minimum Essential Medium (α-MEM) with 10% foetal bovine serum (FBS). The cells were checked periodically to ensure absence of *Mycoplasma* by staining the cells with Hoescht 33258. New cultures were re-established from frozen stock every 3 months. In experiments where cells were grown at different pHe, the cells were maintained in pH-adjusted media. pH-adjusted medium was prepared by mixing α-MEM with 10% FBS, 25 mM HEPES, and the appropriate amount of HCl or NaOH. The medium was allowed to equilibrate in 95% air and 5% CO2 and its pH was repetitively re-adjusted during a one week period.

### Reagents

Cariporide, S3705 and rat-chow containing 0.6% cariporide were supplied by Aventis (Frankfurt, Germany). 5-N-ethyl-N-isopropyl amiloride (EIPA) was obtained from Aldrich (Milwaukee, WI, USA). DIDS, Nigericin and melphalan were purchased from Sigma (Oakville, ON, Canada). 2′7′-bis-(2-caboxyethyl)-5-(and-6)carboxyfluorescein (BCECF) acetoxymethyl ester was purchased from Molecular Probes (Eugene, OR, USA).

### Solutions

Cariporide and S3705 were dissolved in phosphate buffered saline. EIPA was dissolved in 10% DMSO and DIDS was dissolved in distilled water.

Unless otherwise indicated, all solutions were HCO_3_^−^ free. Solution A contained 140 mM NaCl, 5 mM KCl, 5 mM glucose, 1 mM CaCl_2_, 1 mM MgCl_2_, buffered to pH 7.4 with 20 mM MES/Tris. NaHCO_3_ solution contained 25 mM NaHCO_3_, 115 mM NaCl, and other components identical to those in the Solution A; it was prepared and stored without NaHCO_3_, which was added immediately before use. N-Methyl-D-glucamine (NMG) solution was prepared as an iso-osmotic replacement of NaCl; the other components were identical to those described above for Solution A. NH_4_Cl solution contained 15 mM NH_4_Cl and other components identical to the NMG solution. KCl solution contained 20 mM NaCl and 140 mM K^+^ ions.

### Evaluation of pHi and its regulation in cells grown in monolayer

Cells grown as a monolayer on a glass coverslip were exposed to 2 μg ml^−1^ of the acetoxymethyl ester BCECF in serum free α-MEM at 37°C for 30 min. The coverslip was rinsed with PBS and placed into a cuvette using a specially designed holder aligned at an angle of 30° to the excitation beam of a SLM Aminco Bowman Series 2 fluorescence spectrometer. The holder also served as a cap for the cuvette, minimizing the loss of CO_2_. The cells were exposed to excitation beams at 495 nM and 440 nM. The ratio of the fluorescence emitted at 525 nM when excited by the 495 nM beam (pH dependent emission) to that emitted at 525 nM when excited by the 440 nM beam (pH independent emission) was used to calculate pHi. A calibration curve of the fluorescence ratio against pHi was made by placing a coverslip into cuvettes containing nigericin and KCl solution of various pHe (7.4–6.2) (Thomas *et al*, 1979).

The ammonium prepulse method was used to achieve intracellular acidification. The cells were placed in 15 mM NH_4_Cl for 30 min. Intracellular acidification was produced when the cells were transferred to NMG solution free of NaCl and NH_4_Cl (Boron, 1989).

Following acidification, the NMG solution was replaced with Solution A (containing 140 mM NaCl), and the consequent rate of increase in pHi was used to measure the activity of the Na^+^/H^+^ exchanger. The efficacy of cariporide and EIPA in inhibiting this exchanger was evaluated by quantitating Na^+^/H^+^ exchange in the presence of different concentrations of inhibitors. Similarly, the activity of the Na^+^-dependent Cl^−^/HCO_3_^−^ exchanger was assessed by replacing the NMG solution with NaHCO_3_ solution containing 10 μM of EIPA (to inhibit the Na^+^/H^+^antiport). S3705 and/or DIDS were added at different concentrations to compare their inhibitory effects on the exchanger. The combined activity of the pH regulatory mechanisms was measured by replacing the NMG solution with NaHCO_3_ solution. Measurements of change in pHi under various conditions were converted to estimates of H^+^ efflux by using the calculations presented previously (Boron, 1989).

To evaluate possible changes in expression of the exchangers induced by exposure to their inhibitors, cells were grown in the presence or absence of 80 μM cariporide and 40 μM S3705 at different pHe for up to 7 days then seeded on coverslips and allowed to attach overnight before use. The pHi of the cells, and activity of the exchangers was then evaluated as described above.

### Assays of *in vitro* toxicity

The toxicity of cariporide and/or S3705 to cells grown under conditions of different pHe was evaluated by a clonogenic assay. Cells in monolayer were exposed to cariporide (80 μM) and/or S3705 (40 μM) in α-MEM + 10% FBS + 25 mM HEPES buffered to various pHe (7.4–5.9). Control cells were exposed to the solvents used for cariporide and S3705. Following a 24 h incubation period at 37°C in 95% air and 5% CO_2_, the cells were trypsinized, washed and plated in tissue culture dishes. The plates were incubated for 10–14 days and the colonies were stained with methylene blue. Colonies containing at least 50 cells were counted and the surviving fraction was calculated as the ratio of the plating efficiencies of treated and control plates exposed to the same pHe.

### Inhibition of cell proliferation

Cells were seeded into 25 cm^2^ flask and left overnight in α-MEM. Following the overnight incubation, the medium was replaced with medium adjusted to different pH with or without 80 μM cariporide and 40 μM S3705. The pH-adjusted media was changed every 2 days until the end of the 7-day experiment. At 2-day intervals, one flask was selected at random, the cells were detached with 0.1% trypsin, and counted. Growth curves were plotted as a function of pHe in the presence or absence of cariporide and S3705.

## RESULTS

### Ability of cariporide and S3705 to inhibit their respective pHi regulatory mechanisms

**Figure 1:**
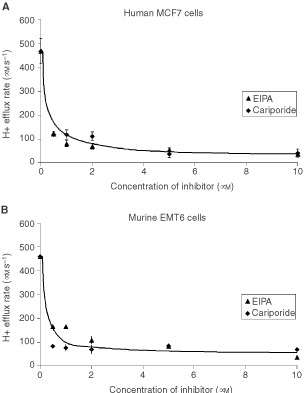
Comparison of the inhibitory effects of EIPA and cariporide on the activity of the Na^+^/ H^+^ antiport (measured by the H^+^ efflux rate in μM s^−1^) for (**A**) MCF7 and (**B**) EMT6 cells. The results indicated are the mean±s.e.m. of at least three experiments.

We assessed the ability of cariporide to inhibit the activity of the Na^+^/H^+^ antiport by measuring H^+^ efflux from cells following intracellular acidification using the ammonium-prepulse method. Our results indicate that cariporide has similar efficacy and potency as EIPA over the range of concentrations that were tested on the MCF7 and EMT6 cell lines (Figure 1

). The efficacy of cariporide to inhibit the Na^+^/H^+^ antiport of MDA-MB231 cells was tested only at a concentration of 5 μM. At a concentration of 5 μM, cariporide was able to inhibit up to 90% of the activity of the Na^+^/H^+^ antiport in each cell line. The experiments were repeated in the presence of 10% FBS, and there was no significant effect of serum on the inhibitory effects of cariporide (data not shown).

**Figure 2:**
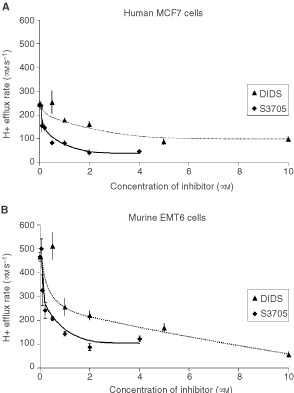
Comparison of the inhibitory effects of DIDS and S3705 on the activity of the Na^+^ dependent Cl^−^/ HCO_3_^−^ exchanger (measured by H^+^ efflux rate in μM s^−1^) for (**A**) MCF7 and (**B**) EMT6 cells. Experiments were performed in the absence of serum. The results indicated are the mean±s.e.m. of at least three experiments.

**Figure 3:**
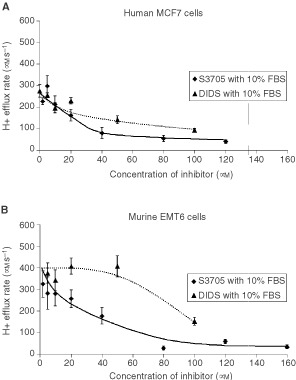
Comparison of the inhibitory effects of DIDS and S3705 in the presence of 10% foetal bovine serum (FBS) on the activity of the Na^+^ dependent Cl^−^/ HCO_3_^−^ exchanger (measured by H^+^ efflux rate in μM s^−1^) for (**A**) MCF7 and (**B**) EMT6 cells. The results indicated are the mean±s.e.m. of at least three experiments.

Similarly, we compared the efficacy and potency of S3705 and DIDS to inhibit the activity of the Na^+^-dependent Cl^−^/HCO_3_^−^ exchanger. In the absence of 10% FBS, S3705 was more effective and more potent than DIDS (Figure 2

). In all three cell lines, up to 80% of exchanger activity was inhibited by 40 μM S3705 (the efficacy of S3705 to inhibit the Na^+^-dependent Cl^−^/HCO_3_^−^ exchanger in MDA-MB231 cells was evaluated only at 40 μM S3705). However, in the presence of 10% FBS, higher concentrations of S3705 were required to obtain equivalent inhibitory effects although this agent was still more effective and more potent than DIDS (Figure 3

).

The contributions of each of the regulatory mechanisms to the recovery of pHi following acidification, and the maximum inhibitory effects from different agents are summarised in Table 1

**Table 1:**
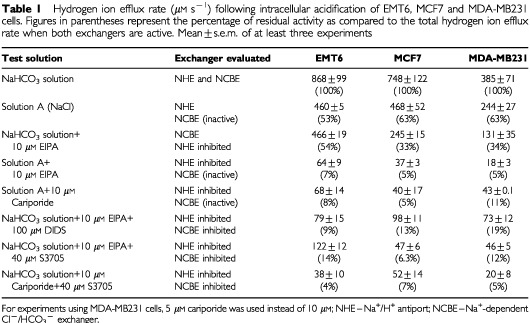
Hydrogen ion efflux rate (μM s^−1^) following intracellular acidification of EMT6, MCF7 and MDA-MB231 cells. Figures in parentheses represent the percentage of residual activity as compared to the total hydrogen ion efflux rate when both exchangers are active. Mean±s.e.m. of at least three experiments

.

### Effects of cariporide and S3705 on the regulation of intracellular pH

**Figure 4:**
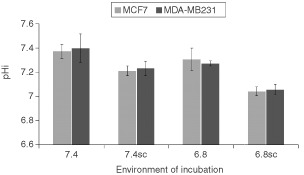
Intracellular pH (pHi) of MCF7 cells (light histograms) and MDA-MB231 cells (dark histograms) following a 24-h incubation period in media adjusted to different pHe (7.4 or 6.8) in the absence or presence of S3705 and cariporide (SC). The results indicated are the mean±s.e.m. of at least three experiments. Student *t*-test indicates that the pHi of the cells incubated in the presence of the inhibitors is lower than the pHi of cells incubated at the same pHe in their absence (i.e. 7.4 *vs* 7.4SC, 6.8 vs 6.8SC) (*P*⩽0.02, both comparisons).

**Figure 5:**
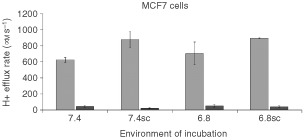
Light histograms: Comparison of the maximum H^+^ efflux rates (measured in μM s^−1^) of MCF7 cells after incubation for 7 days in media adjusted to different pHe (7.4 or 6.8) in the absence or presence of S3705 and cariporide (SC). The agents were not present during evaluation of H^+^ efflux. Dark histograms: Evaluation of H^+^ efflux in the presence of S3705 and cariporide following similar incubation. The results indicated are the mean±s.e.m. of at least three experiments. Student *t*-test indicates that there was a significant difference in the H+ efflux rates of cells incubated at pHe 7.4 *vs* 7.4SC (*P*⩽0.05) and a non-significant trend (*P*⩽0.10) is observed at pHe 6.8.

We measured the pHi of MCF7 and MDA-MB231 cells after they were incubated for either 24 h or 7 days at pHe 7.4 or 6.8, with or without cariporide and S3705 to evaluate changes in pHi (Figure 4

) and proton efflux rate (Figure 5

). Our results indicate that both cell lines maintained pHi at physiological levels (pHi ≅ 7.3–7.4) when they were incubated for 24 h at pHe 7.4 or 6.8 in the absence of the agents. Incubation in cariporide and S3705 at either pHe caused the pHi of the cells to drop and this decrease in pHi was more marked following incubation at pHe 6.8.

Following 7 days of exposure to cariporide and S3705 at pHe 7.4 or 6.8, we observed that the proton efflux rates of MCF7 cells increased slightly in comparison with cells incubated at the same levels of pHe in the absence of the inhibitors (Figure 5). Cells maintained in the absence of the inhibitors at pHe 7.4 and 6.8 maintained the same proton efflux rates. Despite the upregulated proton efflux rates, cariporide and S3705 maintained their activity to inhibit the exchangers in the cells (Figure 5).

### Toxicity of cariporide and S3705 to cultured cells

The results of clonogenic assays indicate that neither compound was toxic to MCF7 or MDA-MB231 cell lines when cariporide (80 μM) and S3705 (40 μM) were added individually or together to cells buffered to pHe in the range of 7.4–5.9 (data not shown).

### Inhibition of tumour cell proliferation by cariporide and S3705

**Figure 6:**
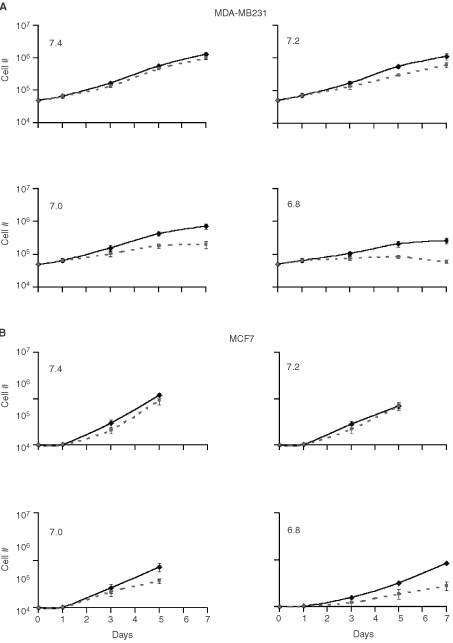
Comparison of the growth rate of (**A**) MDA-MB231and (**B**) MCF7 cells in media adjusted to different pHe (7.4–6.8) in the absence (solid lines) or presence (dashed lines) of (40 μM) S3705 and (80 μM) cariporide (SC). Data for MCF7 cells were plotted only from days 0–5 for pH 7.4–7.0 because the flasks were too confluent for exponential growth beyond day 5. The results indicated are the mean±s.e.m. of five experiments.

The ability of S3705 and cariporide to inhibit the proliferation of MDA-MB231 and MCF7 cells was assessed by evaluating the growth rate of cells at different pHe in the presence or absence of the agents (Figure 6

). As pHe decreased, both cell lines showed decreased growth rates. The growth rates of the cells at pHe 7.4 and 7.2 in the presence of 5 μM S3705 and 10 μM cariporide were not significantly different from the growth rates of the control cells grown in the same pHe. However, MDA-MB231 cells that were incubated with 5 μM S3705 and 10 μM cariporide at pHe 7.0 or 6.8 had a slower rate of growth than control cells at the same pHe (data not shown). In contrast, the growth rates of MCF7 cells at pHe 7.0 or 6.8 in the presence of 5 μM S3705 and 10 μM cariporide did not differ from untreated MCF7 cells grown at pHe 7.0 or 6.8. When these experiments were repeated using higher concentrations of S3705 (40 μM) and cariporide (80 μM), the inhibitory effects of the agents were similar for MDA-MB231 cells (Figure 6A), while MCF7 grown at pHe 7.0 or 6.8 were also inhibited from proliferating as compared to control cells growing in the same pHe (Figure 6B).

## DISCUSSION

Our group and others have tried previously to exploit the acidic microenvironment in solid tumours as a treatment for cancer (Horvat *et al*, 1993; Newell and Tannock, 1989). Although these prior studies showed that inhibiting the regulation of pHi and inducing intracellular acidosis could cause tumour cell death, the therapeutic potential of these strategies was limited by toxicity or by lack of specificity of the agents used.

The present study demonstrates the inhibitory effects of cariporide and S3705 on the Na^+^/H^+^ antiport and the Na^+^-dependent Cl^−^/HCO_3_^−^ exchanger of three malignant cell lines, two of them derived from human breast cancers. Our results suggest that cariporide is as potent and efficient as EIPA while S3705 is more efficient and potent than DIDS. Data from Aventis Pharmaceutical company suggest that a large proportion of S3705 is plasma bound and our results confirm that a higher concentration of S3705 is needed to inhibit Na^+^-dependent Cl^−^/HCO_3_^−^ exchange in the presence of serum. However, S3705 is more effective and potent than DIDS, either in the presence or absence of serum.

Following mild acidification of the cell lines that were tested, Table 1 shows that regulation of pHi was due largely to the combined activity of the Na^+^/H^+^ antiport and the Na^+^-dependent Cl^−^/HCO3^−^ exchanger. Combined application of cariporide and S3705 abolished all but 4–7% of the rate of H^+^ efflux in these cells. Residual H^+^ efflux might be due to incomplete inhibition of the exchangers, or to the activity of other pH regulatory mechanisms including other isoforms of HCO3^−^/Cl^−^ or Na^+^/H^+^ exchangers, H^+^-ATPases and the transport of H^+^ from the cytoplasm into endosomal vesicles.

It is known that the extracellular pH in solid tumours is more acidic (mean values of pHe ≅ 6.9–7.0) than in normal tissues (mean value of pHe ≅ 7.4), and that the pHe in tumours decreases with increasing distance from blood vessels (Helmlinger *et al*, 1997; Wike-Hooley *et al*, 1984; Vaupel *et al*, 1989). Although there may be a small increase in the number of apoptotic cells in regions of low pH, cells have a remarkable ability to withstand extracellular acidity when evaluated by clonogenic assay. Both cariporide (80 μM) and S3705 (40 μM) failed to show toxicity to cells grown in monolayers at doses that inhibited pHi regulation, even at relatively low levels of extracellular pH. This is consistent with previous results for EIPA and DIDS which only become toxic at low pHe if the cells are first acidified by using an ionophore such as nigericin (Newell *et al*, 1992; Yamagata and Tannock, 1996). Thus cariporide and S3705 are unlikely to be directly toxic to cells in solid tumours, despite the tendency of the microenvironment to be acidic; they might however enhance the activity of agents that cause intracellular acidification (Newell *et al*, 1992; Yamagata and Tannock, 1996), but this was not evaluated directly.

It has been shown that intracellular acidosis can inhibit cellular metabolism: low pHi may interfere with the folding and function of proteins such as DNA polymerase and phospho-fructokinase (Bock and Frieden, 1976; Busa and Nuccitelli, 1984). Low pHi, in addition to inhibiting the glycolytic pathway can increase consumption of ATP as the cell activates its H^+^ ATPase to export H^+^ to the extracellular environment or into acidic vesicles. Some investigators have reported that intracellular acidosis could also lead to the activation of the apoptotic cascade (Shrode *et al*, 1997; Park *et al*, 1999; Matsuyama *et al*, 2000), while others have shown that low pHi causes cells to accumulate in G1 phase (Musgrove *et al*, 1987).

To verify the ability of cariporide and S3705 to change the pHi of cells cultured *in vitro* and thus lead to decreased cellular proliferation, we incubated the cells for 24 h at pHe 7.4 and 6.8 in the presence or absence of the agents. Following the incubation period, we observed that cells in media adjusted to pHe 7.4 or 6.8 without the agents maintained a higher pHi than the cells that were incubated at the same pHe with the agents present. This may be due to the accumulation of H^+^ ions that cannot be excreted in the presence of cariporide and S3705. We then tested the ability of cariporide and S3705 to maintain their inhibitory effects on the membrane-based exchange mechanisms. MCF7 cells were incubated for 7 days in media adjusted to different pHe in the presence or absence of the inhibitors. We found that cells grown at pHe 7.4 showed similar H^+^ efflux rates as cells grown at pHe 6.8. Cells incubated for 7 days with cariporide and S3705 showed up-regulated H^+^ efflux rates at both pHe 7.4 and 6.8. The cells were given ample time (3–4 h) to restore their pHi prior to testing their H^+^ efflux rates, thus the increase H^+^ efflux rates are probably not due to increased H^+^ gradients in cells treated with cariporide and S3705. However, when we measured the H^+^ efflux rate in the presence of cariporide and S3705, we found that the agents still provided almost complete inhibition of the exchangers. These results demonstrate the ability of the inhibitors to maintain inhibition of the regulation of pHi, which might lead to the selective inhibition of proliferation of cells growing in acidic conditions.

When we incubated the cells at different pHe (7.4–6.8) in the presence or absence of 10 μM cariporide and 5 μM S3705, concentrations that would effectively inhibit the exchangers, we found that the inhibitors had minimal effects on the growth rate of cells at physiological pHe (7.4 and 7.2). However, the presence of cariporide and S3705 in media adjusted to pHe 7.0 and 6.8 decreased the rate of growth of the cells. Higher concentrations of these agents were needed to inhibit the growth rate of MCF7 cells. These effects might be useful in inhibiting the tumour proliferation of tumour cells that are situated in slightly acidic regions of tumours. Although cells in such regions may have slower rates of proliferation than more proximal cells, (e.g Hirst *et al*, 1982), proliferation of cells from such regions is probably responsible for re-growth of many solid tumours following treatment with radiation or with anti-cancer drugs.

Tumour repopulation, or the proliferation between dose fractions of cells that survive the cytotoxic effects of radiotherapy, is recognized as a probable cause of treatment failure (Withers *et al*, 1988; Maciejewski *et al*, 1989). There is evidence to suggest that following fractionated irradiation applied to murine and human tumours, the repopulation rate of the tumours accelerates with time (Withers *et al*, 1988; Begg *et al*, 1991; Milas *et al*, 1991; Durand, 1997). This accelerated repopulation may be due to an increased growth fraction (Sham and Durand, 1999), to reduced cell loss or to down-regulation of apoptosis (Thames *et al*, 1996). Due to accelerated repopulation, higher doses of radiation or accelerated radiotherapy (or radiation given over shorter total time) may be required to achieve tumour control (Withers *et al*, 1988; Maciejewski *et al*, 1989).

Unlike radiotherapy, where dose fractions are usually given to the patient once a day, patients undergoing chemotherapy most often receive courses of treatment at less frequent intervals, typically every three weeks. This is necessary to allow recovery (by repopulation) of critical normal tissues such as the bone marrow. Repopulation of tumour cells during the longer intervals between courses of chemotherapy is likely to be an important factor that undermines the efficacy of treatment (Milas *et al*, 1991; Davis and Tannock, 2000).

Inhibition of tumour repopulation through the use of selective-selective cytostatic agents given during radiotherapy or between courses of chemotherapy has substantial potential to increase their therapeutic efficacy. Since repopulation of normal tissues is necessary for their recovery, methods to inhibit repopulation must be tumour specific. Radiation will kill selectively aerobic cells in tumours that are likely to be at higher pH while anticancer drugs are also likely to exert preferential cell kill against cells that are close to tumour blood vessels because of higher rates of cell proliferation and/or limited tissue penetration of anti-cancer drugs (Tunggal *et al*, 1999; Tannock *et al*, 2002). Thus agents such as cariporide and S3705 might decrease the rate of cellular proliferation of cells in acidic environments that survive such treatments. Experiments to test the potential of S3705 and cariporide to inhibit repopulation in experimental tumours between cycles of chemotherapy are in progress. These agents have been shown to exert *in vivo* effects to limit myocardial ischaemia; a key question is whether sufficient concentration may be achieved *in vivo* to inhibit the proliferation of cancer cells under the acidic conditions found in solid tumours.
